# Metabolic Clues to Bile Acid Patterns and Prolonged Survival in Patients with Metastatic Soft-Tissue Sarcoma Treated with Trabectedin

**DOI:** 10.3390/metabo13101035

**Published:** 2023-09-26

**Authors:** Gianmaria Miolo, Angela Buonadonna, Simona Scalone, Davide Lombardi, Lara Della Puppa, Agostino Steffan, Giuseppe Corona

**Affiliations:** 1 Department of Medical Oncology, Unit of Medical Oncology and Cancer Prevention, Centro di Riferimento Oncologico (CRO), IRCCS Aviano, 33081 Aviano, Italy; gmiolo@cro.it (G.M.); abuonadonna@cro.it (A.B.); sscalone@cro.it (S.S.); dlombardi@cro.it (D.L.); 2 Unit of Oncogenetics and Functional Oncogenomics, Centro di Riferimento Oncologico (CRO), IRCCS Aviano, 33081 Aviano, Italy; ldellapuppa@cro.it; 3 Immunopathology and Cancer Biomarkers, Centro di Riferimento Oncologico (CRO), IRCCS Aviano, 33081 Aviano, Italy, 33081 Aviano, Italy; asteffan@cro.it

**Keywords:** sarcoma, bile acids, tauroursodeoxycholic acid, glycoursodeoxycholic acid, ursodeoxycholic acid, metabolomics, trabectedin

## Abstract

Metastatic soft-tissue sarcomas (mSTS) encompass a highly heterogeneous group of rare tumours characterized by different clinical behaviours and outcomes. Currently, prognostic factors for mSTS are very limited, posing significant challenges in predicting patient survival. Within a cohort of 39 mSTS patients undergoing trabectedin treatment, it was remarkable to find one patient who underwent 73 cycles of trabectedin achieving an unforeseen clinical outcome. To identify contributing factors to her exceptional long-term survival, we have explored circulation metabolomics and biohumoral biomarkers to uncover a potential distinct host biochemical phenotype. The long-term survival patient compared with the other mSTS patients exhibited a distinctive metabolic profile characterized by remarkably higher levels of ursodeoxycholic acid (UDCA) derivatives and vitamin D and lower levels of lithocholic acid (LCA) derivatives, as well as reduced levels of inflammatory C-Reactive Protein 4 (C-RP4) biomarker. Despite its exploratory nature, this study reveals a potential association between specific bile acid metabolic profiles and mSTS patients’ prognosis. Enhanced clinical understanding of the interplay between bile acid metabolism and disease progression could pave the way for new targeted therapeutic interventions which may improve the overall survival of mSTS patients.

## 1. Introduction

Metastatic soft-tissue sarcomas (mSTS) are a highly heterogeneous group of rare tumours characterized by specific macroscopic, immunohistochemical, cytogenetic, molecular, and metabolic signatures that can significantly impact drug responsiveness to trabectedin. This latter anticancer drug represents an effective second-line treatment option for patients with locally advanced and mSTS since it demonstrated significant improvements in both progression-free survival and overall survival, particularly within the L-type sarcoma group, which includes the leiomyosarcoma (LMS) and liposarcoma (LPS) histotypes [1]. The pivotal clinical study brought attention to a notable subset of patients with LMS and LPS histotypes which received the best clinical benefit from the treatment. Among these, a significant number of patients successfully completed six or more treatment cycles, and remarkably, a few even progressed through an impressive 28 cycles. This intriguing diversity in the clinical benefit duration underscores the substantial variability observed among patients [1], as later supported by subsequent investigations [2,3]. To date, the most remarkable clinical benefit duration achieved through trabectedin treatment for a patient diagnosed with LMS results of 94 cycles [4]. This extraordinary treatment outcome can only be partially attributed to the drug’s mechanisms of action, which alter both the DNA cell nucleus and the tumour microenvironment activity, resulting in its oncostatic effect [5]. Accumulating data suggest that the efficacy of the trabectedin treatment may be attributed, besides tumour histology, also to differences in the overall host molecular and biochemical features [6,7,8,9,10]. In this context, the pivotal role of tumour microenvironmental matrix peptidases and their dynamic tumour interaction may be instrumental in shaping the overall anticancer activity of trabectedin. This further underscores the importance of considering host molecular characteristics as a determinant for the pharmacological outcome [11,12,13,14]. Among the metabolic features, citrulline has been recently found strongly associated with survival outcomes, and it holds the potential to elucidate the substantial prognostic variability observed among mSTS individuals. Variability in treatment response and tolerability can also be influenced by individual differences of drug clearance rates. We recently reported an unexpected distinct inverse relationship between drug clearance rates and treatment response of mSTS [15]. Taking into account a group of mSTS patients, the present investigation focused on a 54-year-old patient with LMS who received an extraordinary clinical benefit from the trabectedin treatment that spanned over 73 cycles. The principal aim of the current study was to research for specific biochemical profiles based on host metabolomic features and classical biohumoral parameters potentially associated with the prolonged disease stability and long-term survival of a patient among an mSTS population who underwent trabectedin treatment. 

## 2. Materials and Methods

### 2.1. Characteristics of Patients

The study considered a total of 39 patients with various mSTS histotypes and clinical characteristics (Table 1). The median age of the patients was 66 years (interquartile range (IQR) 49–72 years). Male patients represented 56% of the total. Thirteen out of 39 patients, either frail for advanced age or previously treated with anthracycline-based regimens, received trabectedin as first-line treatment, while 26 patients underwent second- or third-line treatment with the same drug. Out of the 26 patients who underwent second- or third-line trabectedin treatment, a total of 11 were administered as first-line treatment a combination of non-pegylated doxorubicin and ifosfamide, 8 received an epirubicin and ifosfamide combination, 2 were treated with a combination of gemcitabine and docetaxel, 2 patients received gemcitabine as a monotherapy, 1 patient received a carboplatin and paclitaxel combination, another patient underwent treatment with a combination of vincristine, adriamycin, and ifosfamide, while the remaining patient received a combination of ifosfamide, cisplatin, and doxorubicin. The majority of patients had a high-grade STS (74%). Among these, 15 patients exhibited the same histotype (L-sarcomas) as the index case, while 23 patients had different histotypes including undifferentiated pleomorphic sarcoma (*n* = 4), malignant peripheral nerve sheath tumour (*n* = 3), fibrosarcoma (*n* = 2), myxofibrosarcoma (*n* = 2), chondrosarcoma (*n* = 2), synovial sarcoma (*n* = 2), endometrial stromal sarcoma (*n* = 2), desmoplastic small-round-cell tumour (*n* = 1), carcinosarcoma (1), not-otherwise-specified sarcoma (*n* = 4), and were lumped as “Other-sarcomas”. 

### 2.2. Clinical Journey of Index Case

In July 2013, a 43-year-old woman underwent a large excision of a left thigh neoformation. Histological evaluation revealed a cell morphology characterized by spindle-shaped smooth muscle cells. Immunohistochemical (IHC) analysis indicated positive expression of α-smooth muscle actin (1A4), muscle actin monoclonal antibody (HHF35), desmin, calponin, and Bcl2, while CD34, S100 protein, and epithelial membrane antigen (EMA) were all negative, consistent with an LMS diagnosis. IHC of mismatch repair (MMR) proteins as well as the *NTRK1*, *NTRK2*, *NTRK3*, *FGFR1*, *FGFR2*, *FGFR3*, *ALK*, *BRAF*, *RET*, *ROS1*, and *NRG1* gene fusion analysis was also performed. The sample resulted proficient for the MMR proteins (pMMR) and wild type for all genes analysed. 

**Figure 1:**
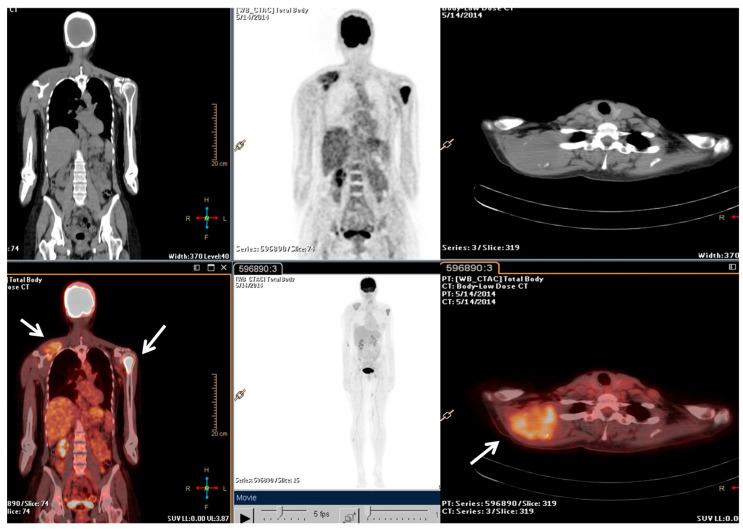
The F-18 Fluorodeoxyglucose (FDG) Positron Emission Tomography/Computed Tomography (PET/CT) performed at baseline of radiotherapy treatment, nine months from the diagnosis. The main tumour lesions are indicated by white arrows.

Following the stage of the disease (pT1b, IA) and grade (G2), a clinical–instrumental follow-up was initiated. Nine months after the initial diagnosis, a PET/CT scan detected a large, metabolically active solid mass involving the right scapula and the periscapular muscles, as well as the involvement of cervical spine segment and the left proximal humerus (Figure 1). 

**Figure 2:**
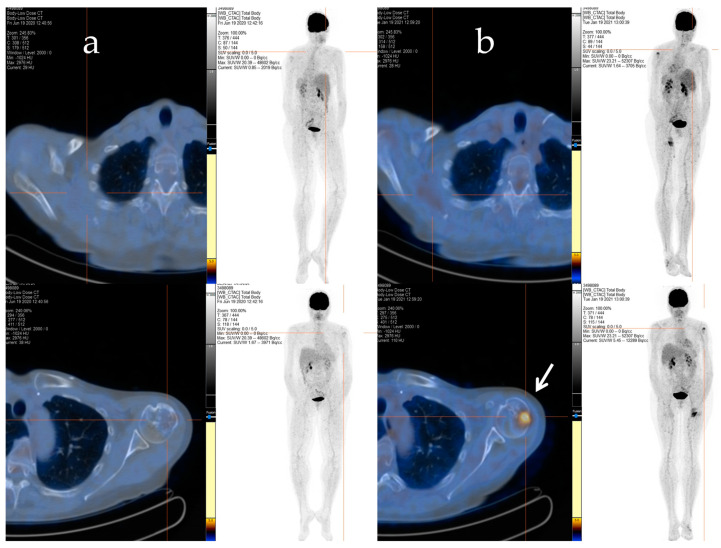
The F-18 FDG PET/CT performed after 48 cycles of trabectedin treatment (**a**) and after the period of trabectedin suspension (**b**). The main tumour lesions are indicated by white arrows.

Afterwards, radiation therapy to the cervical segment (C4–C6) and left humerus (20 Gy/5 fractions) was performed, and a chemotherapy regimen consisting of epirubicin and ifosfamide was started. Despite an initial positive response after six cycles of treatment, signs of bone and soft tissue progression were apparent three months after the chemotherapy ended. Thus, the patient received a second-line treatment with trabectedin (1.5 mg/m^2^ q21), which was administered as a 24 h infusion every 3 weeks. After 48 cycles of treatment, despite the stable disease, the patient decided to discontinue the treatment due to severe fatigue, and a close follow-up was started. The patient maintained stable disease until January 2021 when a PET/CT scan revealed an increased uptake in the left proximal humerus (Figure 2a,b), right scapula, sternal manubrium, right iliac bone, and ipsilateral acetabulum.

**Figure 3:**
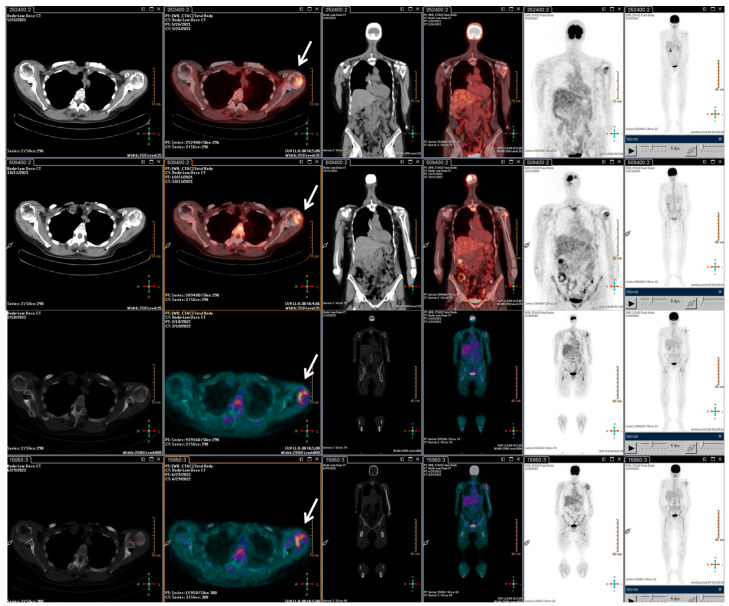
F-18 FDG PET/CT performed after the radiotherapy retreatment. The main tumour lesions are indicated by white arrows.

With the rising of the disease, trabectedin treatment was resumed and continued for an additional 21 cycles until January 2022, when a PET/CT scan revealed a slight metabolic progression in the left humeral head. Thus, in March 2022, radiotherapy treatment on the left humeral head was completed (20 Gy/5Fractions), and, considering the stable disease revealed in July 2022, the trabectedin administration was continued until February 2023 (Figure 3). 

Overall, the patient underwent a total of 73 cycles of trabectedin. Subsequently, due to disease progression in the left humerus, the patient underwent surgical treatment with prosthetic grafting. Despite the successful procedure, the patient experienced a rapid clinical decline characterized by a lung impressive progression disease, which led to her death shortly thereafter.

### 2.3. Serum Metabolomics Analysis 

The serum samples of patients were subjected to profiling for 55 free amino acids and derivatives using a modified version of the method described by Prinsen et al. [9,16]. Additionally, serum was profiled for 15 bile acids (BAs), including two primary BAs (cholic acid (CA), chenodeoxycholic acid (CDA)), three secondary BAs (deoxycholic (DCA), lithocholic (LCA), and ursodeoxycholic acids (UDCA)), and 10 taurine or glycine-conjugated derivatives. High-performance liquid chromatography coupled with an Agilent 1290 Infinity II binary pump and an Ultivo triple quadrupole mass spectrometer (Agilent Technologies, Santa Clara, CA, USA) equipped with an electrospray ionization source (ESI) was used for the profiling. Intra-assay and inter-assay variability measures for each investigated BA were below 15%.

Additionally, the study considered 30 biohumoral parameters including: White Blood Cells (WBC), Red Blood Cells (RBC), Hemoglobin (Hb), Hematocrit (HCT), Platelets (PTL), Neutrophils (Ns), Lymphocytes (Ls), Monocytes (Ms), Eosinophils (Es), Basophils (Bs), Glucose, Creatinine, Urea, Uric Acid, Aspartate aminotransferase, Alanine transaminase, Gamma-glutamyltransferase, Albumin, Alkaline Phosphatase, Lactate Dehydrogenase, Total Bilirubin, Conjugated Bilirubin, Total Protein, Sodium, Kalium, Chlorum, Calcium, Fibrinogen, Prothrombin time, and activated partial thromboplastin time.

The matrix dataset consisting of 39 patients characterized by 107 clinical (7), metabolomic (70), and biohumoral (30) variables was analysed by unsupervised multivariate principal component analysis (PCA) in order to identify potential data cluster and patient outlier.

## 3. Results

**Figure 4:**
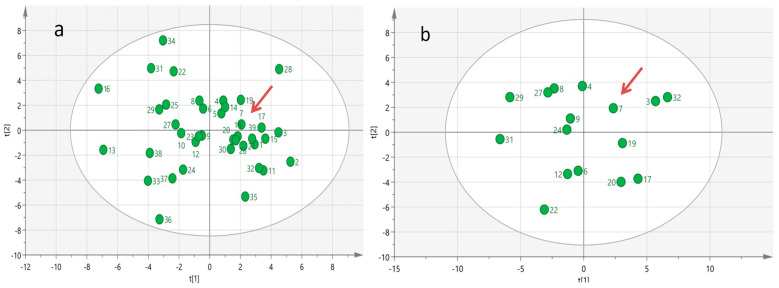
Multivariate Principal Component Analysis (PCA) generated from serum metabolomics profiles of all 39 mSTS patients (**a**) and from 16 more homogeneous mSTS patients selected by L-sarcomas histotype (**b**). The red arrows indicate the case index.

In an attempt to reveal specific biochemical–biohumoral phenotypic characteristics that contributed to the long-term survival achieved by the index case, we surveyed a comprehensive baseline targeted metabolomics profile analysis which encompasses 70 serum metabolites and 30 biohumoral parameters across all groups of mSTS patients. Multivariate PCA analysis of the serum metabolomics profiled data did not reveal any evidence of outlier metabolomic profiles among the patient population investigated (Figure 4a), even in the subgroup of mSTS with the same histotype (Figure 4b), likely due to the great inter-patient metabolomic variability which may mask distinctive metabolic patterns.

**Figure 5:**
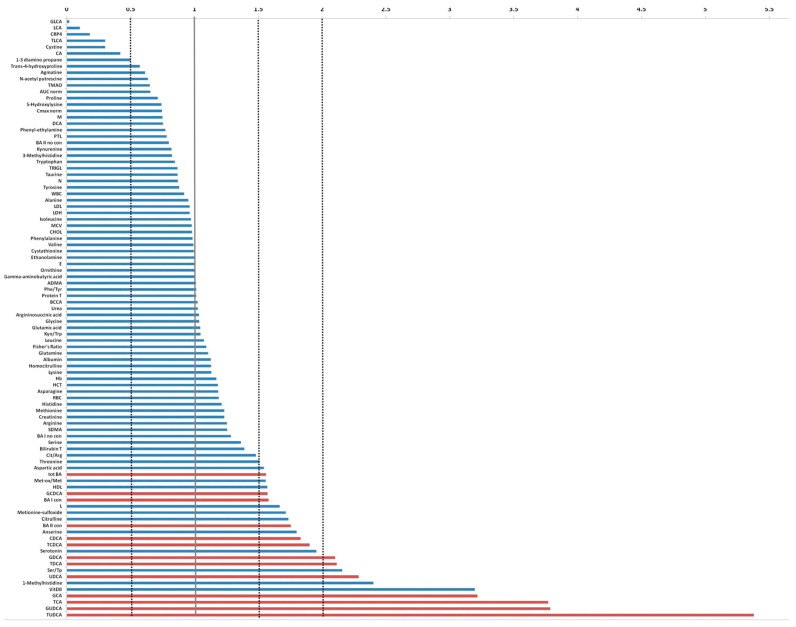
Fold change plot of serum metabolites and biohumoral parameters of the index case compared with the median values achieved from the remaining 38 mSTS patients. In red are indicated the bile acids that exceeded at least 1.5 times the average value.

Comparing the serum level of metabolites investigated and the biohumoral variables of index case with the median values derived from 19 mSTS patients treated with second-line trabectedin, higher levels (more than two folds) of tauroursodeoxycholic acid (TUDCA), glycoursodeoxycholic acid (GUDCA), taurocholic acid (TCA), glycocholic acid (GCA), vitamin D, 1-methylhistidine, ursodeoxycholic acid (UDCA), Ser/Tp ratio, taurodeoxycholic acid (TDCA), and glycodeoxycholic acid (GDCA) were found, whereas lithocholic acid (LCA), glycolithocholic acid (GLCA), and taurolithocholic acid (TLCA) as well as C-Reactive Protein 4 (C-RP4) and Cystine levels were remarkable lower (Figure 5). 

When the comparison involved the group of 25 patients who received trabectedin as second- or third-line treatment, the index patient showed similar fold changes patterns. Analogously, comparing the index case with the 13 patients who underwent first-line trabectedin treatment, a remarkably higher level of vitamin D, TUDCA, GUDCA, UDCA, GCA, taurodeoxycholic acid (TDCA), and chenodeoxycholic acid (CDCA) was detected. Conversely, LCA, GLCA, and TLCA, as well as C-RP4 levels, were notably reduced.

Interestingly, in the index case, the lymphocyte-to-monocyte ratio (LMR) was approximately 2.5 times higher than the median value observed in the 38 mSTS cohort patients, while the neutrophil-to-lymphocyte ratio (NLR) as well as the platelet-to-lymphocyte ratio (PLR) resulted 0.5 times lower. However, these biomarkers were not found strictly associated with the remarkable outcome of the index case since other patients with lower overall survival had similar values.

**Figure 6:**
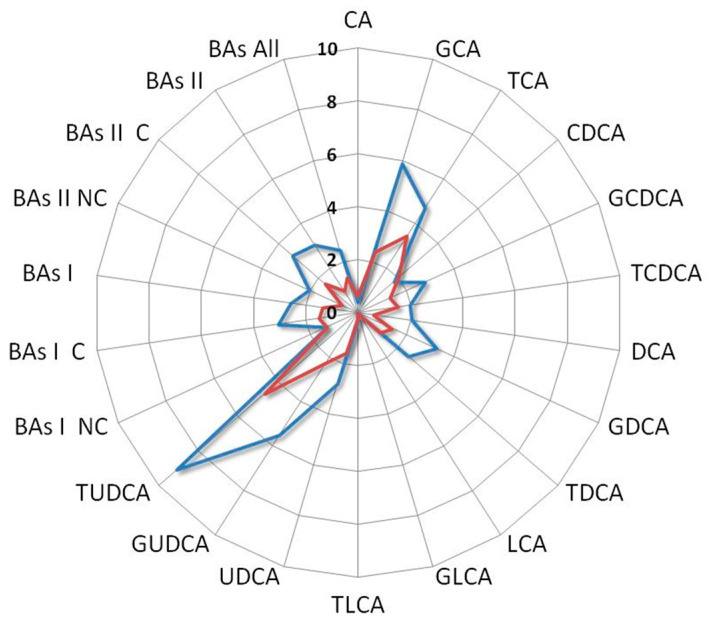
Radar plot of the BAs fold change levels of the index case compared with median levels of mSTS patients. All patients underwent second-line chemotherapy and were stratified by histotype as L-sarcomas (blue line) and Other-sarcomas, (red line). Cholic acid (CA), glycocholic acid (GCA), taurocholic acid (TCA), chenodeoxycholic acid (CDCA), glycochenodeoxycholic acid (GCDCA), taurochenodeoxycholic acid (TCDCA), deoxycholic acid (DCA), glycodeoxycholic acid (GDCA), taurodeoxycholic acid (TDCA), lithocholic acid (LCA), glycolithocholic acid (GLCA), taurolithocholic acid (TLCA), ursodeoxycholic acid (UDCA), glycoursodeoxycholic acid (GUDCA), tauroursodeoxycholic acid (TUDCA), non-conjugated primary bile acids (BAs I NC), conjugated primary bile acids (BAs I C), total primary bile acids (BAs I), non-conjugated secondary bile acids (BAs II NC), conjugated secondary bile acids (BAs II C), total secondary bile acids (BAs II), total bile acids (BAs).

Among all the comparisons, a distinct metabolomics profile appears clearly emerged associated with the enhanced survival of the index case. In particular, such a profile is characterized by elevated levels of vitamin D, UDCA derivatives, while conversely, it exhibits a reduction in LCA derivatives and C-RP4 biomarker (Figure 6). 

**Figure 7:**
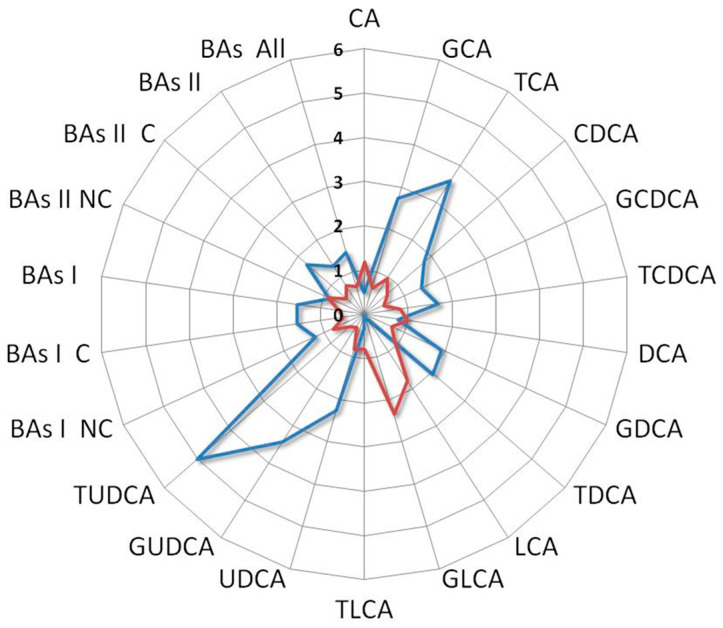
Ratio between BAs levels of the index case with the median values of L-sarcomas patients who underwent second-line treatment (blue line) compared with that derived from an L-sarcomas patient who achieved poor benefit from treatment (red line). Notice the distinct profiles: the former is characterized by high levels of UDCA and its derivatives and low levels of LCA and its derivatives, while the latter is marked by elevated levels of LCA and its derivatives and low levels of UDCA and its derivatives. Cholic acid (CA), glycocholic acid (GCA), taurocholic acid (TCA), chenodeoxycholic acid (CDCA), glycochenodeoxycholic acid (GCDCA), taurochenodeoxycholic acid (TCDCA), deoxycholic acid (DCA), glycodeoxycholic acid (GDCA), taurodeoxycholic acid (TDCA), lithocholic acid (LCA), glycolithocholic acid (GLCA), taurolithocholic acid (TLCA), ursodeoxycholic acid (UDCA), glycoursodeoxycholic acid (GUDCA), tauroursodeoxycholic acid (TUDCA), non-conjugated primary bile acids (BA I NC), conjugated primary bile acids (BA I C), total primary bile acids (BAs I), non-conjugated secondary bile acids (BAs II NC), conjugated secondary bile acids (BAs II C), total secondary bile acids (BAs II), total bile acids (BAs).

Interestingly, the BAs fold change profile of the index case compared with a patient who did not receive any benefit from the treatment showed contrasting trends. Indeed, the first one was characterized by an increase in UDCA and its derivatives along with a decrease in LCA and its derivatives, while the second one showed an increase in LCA and its derivatives and a reduction in UDCA and its derivatives (Figure 7). 

**Figure 8:**
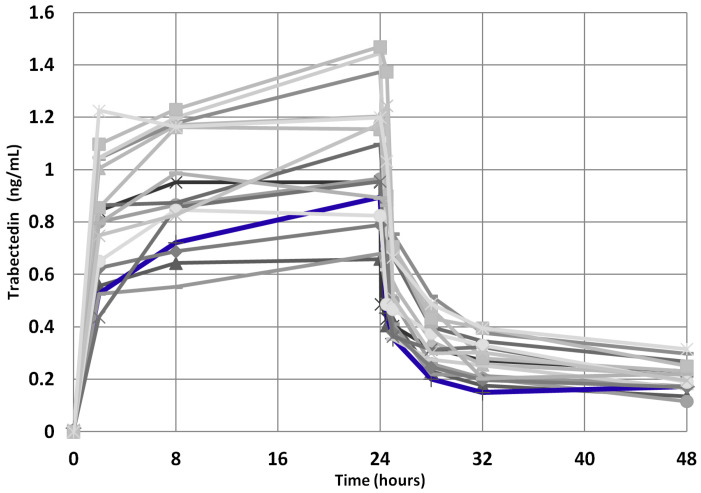
Trabectedin pharmacokinetics profile for the 39 mSTS patients (gray lines). The index case is highlighted (blue line).

Moreover, since the trabectedin pharmacokinetics could have played a role in achieving a so prolonged clinical benefit, we compared the trabectedin pharmacokinetics parameters of the index case with those of the other mSTS patients, but no significant difference in AUC0-48 h, AUC/dose, Cmax, and Cmax/dose was found (Figure 8), these parameters being within the IQR2. Interestingly, the index case showed an AUC max lower than median value derived from both L-sarcomas and Other-sarcomas patients, indicating good drug metabolism capability.

## 4. Discussion

The median life expectancy of mSTS patients treated with trabectedin observed across the clinical studies is approximately 12 months [1,2,17,18]. However, a significant variability in the inter-patient outcomes exists [1,2,17,18], indicating that a subset of patients experienced significantly greater benefits from trabectedin treatment as the patient reported in this investigation who, along her clinical journey, received a remarkable sequence of 73 cycles of trabectedin yielding an exceptionally prolonged disease stability. In order to find a biochemical explanation of this extraordinary outcome, we conducted an in-depth investigation into the differences in the blood circulatory metabolomics and biohumoral blood profile of this patient compared to a group of mSTS patients. 

Within the cohort of patients investigated, the targeted metabolomics analysis revealed for the long-term survival index case a distinctive metabolic profile. This latter was characterized by a notable increase in the secondary BAs levels, including UDCA, TUDCA, and GUDCA, associated with low levels of LCA and its derivatives as compared with those observed in the mSTS population. This specific metabolic feature seems to be associated with an improved survival outcome and, in particular, suggests a potential role of both UDCA and its derivatives as well as LCA and its derivatives in development and progression of mSTS. This result seems to suggest a complex interaction between the host BAs metabolism and the tumour development which, in turn, have the potential to result in significant and meaningful prognostic implications [6,19].

BAs are cholesterol-derived molecules primarily known for their role in metabolism and absorption of dietary fats. They can be classified into two major groups: primary and secondary BAs. Primary BAs, CA and CDCA, are synthesized in the liver and can be conjugated with the amino acids glycine or taurine to form bile salts [20,21]. Secondary BAs, such as deoxycholic acid (DCA), LCA, and UDCA, are produced through bacterial metabolism in the intestinal tract and are transported to the liver via the enterohepatic circulation. A small fraction of the BAs can deviate from this pathway and enter into the systemic circulation instead of being directly transported to the liver, thus to be detected in the serum [21,22,23].

In addition to their role in dietary fat metabolism, BAs are recognized for their diverse effects achieved through the activation of various signaling pathways and their interaction with specific cellular receptors such as the farnesoid X receptor (FXR), that not only regulates the synthesis and transport of BAs, but also exerts a significant influence over a wide range of cellular metabolic functions involved in cell growth [24,25]. Additionally, BAs can activate other receptors, such as the G-protein-coupled BA receptor 1 (GPBAR1), also known as the Takeda G-protein receptor 5 (TGR5), vitamin D Receptor (VDR), influencing multiple processes, including inflammation, cell proliferation, apoptosis, metabolism, and immune responses [25,26,27,28,29]. 

The distinguishing trait of the circulatory levels of BAs as observed in the long-term survival patient may unveil the extensive pleiotropic effects of BAs across a wide spectrum of physiological and pathological processes, including their impact on growth control and development of sarcomas as reported for various tumour diseases [30,31]. In hepatocellular carcinoma (HCC), increased levels of BAs, such as CDCA and DCA, have been associated with tumour growth, angiogenesis, and metastasis while, in colorectal cancer, secondary BAs, such as DCA, have been linked to heightened increased inflammation, DNA damage, and promotion of tumour cell survival [22,25,32,33,34]. Conversely, other studies have suggested a protective effect of certain BAs, such as UDCA, in specific cancers, such as breast and prostate cancer [35,36,37], by inhibiting cell proliferation and inducing apoptosis [38,39,40]. In this complex context, the relatively high levels of secondary BAs, such as UDCA derivatives, observed in this surprising long-term survival patient may suggest a potential protective host mechanism by controlling tumour growth. Specifically, the enhanced levels of UDCA derivatives associated with high levels of vitamin D and low levels of C-RP4 protein might reflect a less inflammatory tumour microenvironment. Interestingly, the index case displayed elevated levels of TCA and GCA, which have recently gained attention for their interaction with FXR to modulate inflammation. Through the FXR activation, they inhibit pro-inflammatory cytokine production, such as interleukin-1β and tumour necrosis factor-alpha, while promoting anti-inflammatory factors reduce the recruitment and activation of inflammatory cells. Furthermore, the FXR-TCA/GCA interaction has been linked to the regulation of gut microbiota composition, which has a pivotal role in immune system modulation and inflammation control [41,42]. To support the inflammatory hypothesis, we can draw upon the data related to the LMR of the index case. Although not directly linked to survival, these data suggest that trabectedin may act to regulate inflammation by decreasing the overall number of monocytes, subsequently reducing the production of pro-inflammatory cytokines [43,44,45,46,47].

Although the circulatory level of BAs can be determined by multiple factors such as diet, genetics, and disease states for secondary BAs, gut microbiota activity may exert a deep modulation of overall composition of the circulating BAs pool, which may be strongly dependent by the reserve of specific bacteria [48,49]. For instance, certain BAs, such as UDCA, TUDCA, and GUDCA, have been reported to exhibit protective effects on gut microbial communities by promoting the growth of beneficial bacteria, thus maintaining a balanced microbiota [50,51]. This bidirectional relationship between BAs and gut microbiota could be responsible for high levels of UDCA and its derivatives detected in the long-term survival patient and, consequently, for her surprising clinical outcome to the trabectedin treatment. Indeed, UDCA is exclusively produced in the gut microbiota by several species of bacteria such as *Clostridium baratii*, *Clostridium absonum*, as well as some strains of *Eubacterium* and certain species of *Ruminococcus* [51,52,53,54,55,56,57,58]. Moreover, recent studies have suggested that certain strains within the bacteroides genus may carry the enzyme capacity for 7β-dehydroxylation and UDCA production [55]. Thus, for the long-term survival patient who presented very elevated levels of secondary BAs, such as UDCA and its conjugated derivatives TUDCA and GUDCA, can be hypothesized a strong bidirectional interplay between the gut microbiota and the host metabolism [59], which may impact the disease control during trabectedin treatment. The index case showed a trabectedin exposition superimposable to that observed in the other mSTS patients; however, it cannot exclude a synergic interaction between UDCA derivatives and the antitumoural effect of trabectedin that may explain the remarkable pharmacological outcome observed.

Conversely to TUDCA, high levels of LCA may have potential cytotoxic and genotoxic effects for liver cholestatic injury, carcinogenesis, and increased inflammation and disease progression, especially in the intestine [25,60,61]. It was remarkable that in the serum/plasma of the long-term survival patient, the level of these LCA derivatives was particularly low compared with the other mSTS patients, while the level of vitamin D was high. Interestingly, LCA has been shown to interfere with VDR activity, a nuclear receptor that in liver and colon cell systems binds to calcitriol [62,63,64,65,66], promoting an anti-inflammatory immune response and enhancing immune surveillance against cancer cells [67,68,69]. The low level of LCA may lead to the normal activation of VDR through its natural ligand that has been associated with anti-proliferative effects in various cancer types [70] by the control of cell cycle regulators, induction of apoptosis, and inhibition of angiogenesis [63,64]. Moreover, the VDR activation due to high vitamin D concentration may induce the expression of CYP3A, a cytochrome P450 enzyme that has been proved to detoxify LCA in the liver and intestine, contrasting its proliferative effects on cancer cells [62,71]. Therefore, in mSTS, the presence of low serum levels of LCA may have anti-proliferative effects mediated by VDR activation through the high level of vitamin D circulation, as observed in the long-term survival patient. In addition, there is emerging evidence that a high level of vitamin D may also have an impact on the immune microenvironment, which may potentially contribute to the antitumour immune responses against tumour cells, potentially affecting tumour growth and progression [68,72]. 

Although this exploratory study showed specific metabolic clues associated with mSTS long survival, it presents some limitations. Firstly, the sample size was limited and characterized by a high heterogeneity, with over half of the patients with other sarcoma histotypes. Secondly, recruiting patients who had undergone many cycles of trabectedin, such as in the index case, posed a significant challenge, thereby limiting the ability to make meaningful comparisons between groups of comparable size to confirm and properly validate these observations.

## 5. Conclusions

This hypothesis-generating investigation highlighted that a specific serum metabolic profile consisting of high levels of UDCA and its conjugates, along with a high level of vitamin D, low levels of LCA and its derivatives, together with C-RP4, may be strongly associated to mSTS long-term survival. These findings suggest the need for further metabolic exploration to better decipher the intricate interactions between BAs derivatives, microbiome, and cancer cell metabolism and to establish the effective pivotal role of BAs in the development and progression of the STS. 

## Figures and Tables

**Table 1:** Clinical characteristics of the patients.

Gender	Number (%)
Male	22 (56%)
Female	17 (44%)
**Median Age**	66 (IQR 49–72)
**PS**	
0	20 (51%)
1	17 (44%)
2	2 (5%)
**Median BMI**	27 (IQR 22.7–31.9)
**Histology**	
L-sarcomas	16 (41%)
Other-sarcomas	23 (59%)
**Grading**	
G1	1 (2,5%)
G2	9 (23%)
G3	29 (74.5%)
**Chemotherapy line**	
First line	13 (33%)
Second line	20 (51%)
Third line	6 (16%)

## Data Availability

The data presented in this study are available on request from the corresponding author. The data are not publicly available due to subject privacy restrictions.
